# The Association Between Expanded ACEs and Behavioral Health Outcomes Among Youth at First Time Legal System Contact

**DOI:** 10.1007/s10802-022-01009-w

**Published:** 2022-12-24

**Authors:** Johanna B. Folk, Megan Ramaiya, Evan Holloway, Lili Ramos, Brandon D. L. Marshall, Kathleen Kemp, Yu Li, Eraka Bath, Daphne Koinis Mitchell, Marina Tolou-Shams

**Affiliations:** 1 Department of Psychiatry and Behavioral Sciences, University of California, San Francisco, San Francisco, CA USA; 2 Department of Epidemiology, Brown University School of Public Health, Providence, RI USA; 3 Department of Psychiatry and Human Behavior, The Warren Alpert Medical School of Brown University, Providence, RI USA; 4 Department of Psychiatry and Biobehavioral Sciences, University of California, Los Angeles, Los Angeles, CA USA; 5 Department of Pediatrics, Brown University, Providence, RI USA

**Keywords:** Adverse childhood experiences, Juvenile justice, Child welfare, Substance misuse, Psychopathology

## Abstract

A growing body of literature has documented high rates of adverse childhood experiences (ACEs) and their effects on behavioral health among adolescents impacted by the juvenile legal system. Most research with justice-impacted youth assesses the ten standard ACEs, encompassing abuse, neglect, and household dysfunction. This body of work has largely ignored the five expanded ACEs which assess social and community level adversity. Justice-impacted youth commonly experience expanded ACEs (racial discrimination, placement in foster care, living in a disadvantaged neighborhood, witnessing violence, bullying), and inclusion of these adversities may enhance predictive utility of the commonly used ACEs score. The current study examined the prospective impact of total ACEs (standard and expanded) on alcohol and cannabis use, substance-related consequences, and psychiatric symptoms during the year following first ever contact with the juvenile court. Results indicate justice-impacted youth experience multiple expanded ACEs prior to first court contact. The expanded ACEs did not predict any of the behavioral health outcomes assessed, over and above the standard ACEs. Inclusion of expanded ACEs in the standard ACEs score may not increase utility in identifying prospective behavioral health outcomes among youth in first time contact with the juvenile legal system.

## Introduction

Adolescents in contact with the juvenile legal system (i.e., justice-impacted youth; JIY) commonly experience multiple adverse childhood experiences (ACEs), placing them at elevated risk for behavioral health treatment needs and continued legal system involvement (Folk et al., 2021). A recent systematic review (Folk et al., 2021) found eight unique studies (reported in 40 articles) documenting high rates of ACEs among JIY, as well as significant associations between ACEs and multiple adverse behavioral health and legal outcomes. In our own study of JIY at time of first contact with the juvenile court (Authors, 2021), those who experienced a greater number of standard ACEs were significantly more likely to use alcohol and report elevated posttraumatic stress symptoms 12 months later. However, standard ACEs were unrelated to cannabis use, substance-related consequences, or internalizing and externalizing symptoms.

The limited predictive utility of the ACEs score among JIY may be due to failure to consider the impact of the expanded ACEs, which adds the five ACEs of racial discrimination, placement in foster care, living in a disadvantaged neighborhood, witnessing violence, and bullying (Cronholm et al., 2015). The ACEs framework was developed with predominantly white, privately insured, middle to upper-middle class adults (e.g., Felitti et al., 1998), and recent evidence suggests the standard ACEs are neither an equitable nor accurate predictor of mental health among all adolescent subpopulations. Research consistently documents disproportionate ethnoracial minoritized youth contact with the legal system (Fielding-Miller et al., 2020; Tolou-Shams et al., 2020) and significant inequities in health outcomes and healthcare access for ethnoracial minoritized youth (Williams et al., 2019). Although studies with non-JIY document higher rates of ACEs among Black and Hispanic adolescent girls than white adolescents (Cohen & Choi, 2022), studies of JIY show that white youth tend to experience a greater number of ACEs than Black and Hispanic JIY (Baglivio & Epps, 2016; Wolff et al., 2018). Ethnoracial minoritized JIY may be particularly likely to experience the five expanded ACEs, particularly racial discrimination and placement in foster care (due to disproportionately high rates of foster care placement, particularly for Black and Indigenous youth, driven by structural racism; Wildeman & Emanuel, 2014). Almost no attention has been paid to the prevalence or cumulative effects of experiencing expanded ACEs among JIY, a disproportionate number of whom are from minoritized, ethnoracial groups. The current study aimed to fill this gap in the literature by examining the impact of the expanded ACEs on future behavioral health outcomes among adolescents in first-time contact with the juvenile court.

## Adverse Childhood Experiences

Adverse childhood experiences, which encompass three broad categories of *abuse* (physical, emotional, sexual), *neglect* (physical, emotional), and *household dysfunction* (caregiver substance use, mental illness, divorce or separation, incarceration, domestic violence), are a significant public health concern. The epidemiological impact of ACEs on health in the United States (U.S.) was first documented in the mid-1990s in the context of a large, retrospective, population-based study, revealing a high prevalence of ACEs among adults, patterns of multiple co-occurring ACEs in households, and a dose-dependent relationship between ACEs and a broad range of health consequences (Anda et al., 2010; Felitti, 2002; Felitti et al., 1998). Although groundbreaking, this early ACEs research was limited by its focus on predominantly white, privately insured, middle to upper-middle class adults (e.g., Felitti et al., 1998).

Subsequent studies have examined the impact of ACEs on behavioral health among more diverse samples of youth. One means of understanding the impact of the standard ACEs on behavioral health is through ecodevelopmental theory (Szapocznik & Coatsworth, 1999), an extension of Bronfenbrenner’s (1986) ecological model of human development (i.e., micro-, meso-, exo- and macro-system influences on behavior) that provides a multi-level framework to understand interacting risk and protective factors for adolescent behavioral health. The standard ACEs address the micro-(individual; e.g., experiencing abuse) and meso- (family; e.g., domestic violence, divorce) system levels of influence, which can include traumatic or potentially traumatic events or experiences that may interact to impact mental health later in life. Studies examining the impact of ACEs on behavioral health among diverse samples of youth have found strong associations between total number of ACEs and poor mental health symptoms (Chatterjee et al., 2018; Schilling et al., 2007), including internalizing (e.g., depression and anxiety) and externalizing symptoms (Cecil et al., 2017; Elmore & Crouch, 2020; H. Y. Lee et al., 2020). Differential associations between individual ACEs and domains of ACEs (e.g., abuse, neglect, household dysfunction) and psychiatric symptoms have also been examined (Cecil et al., 2017; Negriff, 2020). ACEs have also been linked to increased risk of youth substance use (Benedini & Fagan, 2020; Chatterjee et al., 2018; Scheidell et al., 2018).

Some studies suggest, however, that the standard 10 ACEs may not completely capture associations between childhood adversity and youth health (Wade et al., 2016). Of note, in one cross-sectional survey of adolescents (Afifi et al., 2020), peer victimization increased risk of youth substance use, above and beyond the standard ACEs, highlighting the potential utility of considering additional forms of adversity into the original ACEs. In an additional nationally representative study (Finkelhor et al., 2015), nearly one-quarter of youth (age 10–17 years) had witnessed violence in their family or community in the past year, suggesting witnessing violence should also be included under the ACEs umbrella. Black youth describe racial discrimination, community violence, poverty, and interactions with the legal system as salient sources of stress not captured by the standard ACEs (Wade et al., 2014), and evidence shows that racial discrimination is significantly associated with standard ACEs as well as internalizing problems among Black youth (Bernard et al., 2021). Further, researchers caution that the standard ACEs are not equally calibrated across racial and ethnic groups when predicting mental health outcomes and may lead to erroneous referrals for Black males and Hispanic females in particular (Cohen & Choi, 2022). These calls for expansion of the ACEs align with the ecodevelopmental exo-level (extra-familial; e.g., community violence, peer bullying) contexts posited to influence developmental processes, including behavioral health.

## Adverse Childhood Experiences Among Justice-Impacted Youth

A growing body of research has examined the impact of ACEs among JIY, a group of youth for whom childhood adversity is nearly ubiquitous. In a prevalence study of 64,329 JIY, less than 3% experienced zero ACEs and half reported four or more ACEs; rates were significantly higher among girls, who experienced on average 4.29 ACEs compared to 3.48 for boys (Baglivio et al., 2014). According to a recent systematic review on ACEs among JIY in the U.S. (Folk et al., 2021), research has most commonly documented links between ACEs and delinquency outcomes, such as recidivism (e.g., Graf et al., 2021). Studies among JIY in the U.S. have also shown significant, positive associations between ACEs and psychiatric symptoms (Baglivio et al., 2017; Clements-Nolle & Waddington, 2019; Craig et al., 2019; Authors, 2021; J. A. S. Lee & Taxman, 2020; P. Logan-Greene et al., 2017; Patricia Logan-Greene et al., 2020; Muniz et al., 2019; Perez et al., 2018) and substance use (Authors, 2021; Weber & Lynch, 2021). Studies have been mixed regarding the impact of ACEs on trauma symptoms; high-quality studies examined in a systematic review showed no evidence that higher ACEs scores predict trauma symptoms (Malvaso et al., 2021). In one study that broadened standard ACEs to include parental and/or guardian death, peer victimization, witnessing community violence, and family experiences of homelessness, JIY with co-occurring psychological and substance use difficulties reported significantly more ACES relative to those with one or no behavioral health concerns (Lensch et al., 2021). It is possible inclusion of the expanded ACEs increased predictive utility of the ACEs score in this study, suggesting the need for further research in this area among JIY.

## Current Study

The aim of the current study was to examine the prospective association between expanded ACEs and behavioral health outcomes (i.e., psychiatric symptoms, substance use, consequences of substance use) for JIY at time of first contact with the juvenile court. Specifically, we aimed to answer the question of whether *number of expanded ACEs predicts psychiatric symptoms, substance use, and substance use consequences at 12-month follow-up, above and beyond the number of standard ACEs,* in a sample of JIY and their caregivers. It was hypothesized that, even when accounting for standard ACEs, JIY who reported a greater number of expanded ACEs would exhibit greater psychiatric symptoms, substance use, and consequences of substance use. Given that racial and ethnic minoritized youth are often subject to overcriminalization of their behavior, which contributes to disproportionate minority contact in the juvenile legal system (Rovner, 2014), we also explored racial and ethnic differences in the prevalence of ACEs and accounted for ethnicity in multivariate models. Specifically, we aimed to answer the question of whether there are racial and ethnic differences in the prevalence of expanded ACEs. We highlight that race is not a biological variable, but rather a social and political construct often used as a proxy for the impact of structural racism and inequality. The current investigation holds this premise in mind.

## Positionality Statement

The authors recognize our own backgrounds and experiences influence our perspectives on this topic. The authors represent a range of intersecting identities based on race, ethnicity, sex, gender, sexual orientation, disability status, and immigration status. Several authors also have lived experience with childhood adversity and the legal system, either through their own interactions with law enforcement or through family members in the carceral system. We represent a range of professional backgrounds, including clinical psychologists, psychiatrists, and an epidemiologist. Our shared interest in the current investigation stems from: (1) clinical experience with assessing and treating the effects of childhood adversity among justice-impacted youth; (2) recognition that ACEs screenings are being prematurely implemented in clinical settings without sufficient research to support their clinical use; and (3) an understanding that we as researchers have a responsibility to investigate and document the multi-level factors impacting youth who come into contact with the justice system in order to inform anti-racist and trauma-responsive practices within our legal and clinical systems.

## Method

### Participants and Procedure

Youth and caregivers were recruited from a large family court in the northeastern U.S. Those eligible to participate in the larger study: (1) were between the ages of 12 and 18 years; (2) had a first-time, open status (e.g., truancy, curfew, alcohol use) and/or delinquent (e.g., assault, breaking and entering) petition filed within the past 30 days; (3) had no prior history of court involvement; (4) had no significant cognitive impairment; and (5) had a caregiver who lived in the same household with them for at least six months prior to enrollment. All youth resided in the community at point of enrollment. Study flyers were distributed to potential participants alongside their court date notification letters. Research assistants met with youth and families at their first court appointment to discuss potential study participation and complete a private, separate screening with those interested in participating. Eligible and interested youth and families met with research staff outside of the court setting (e.g., at home, private community space, research lab) to provide assent and consent for study participation. Girls were intentionally oversampled and those with open delinquency and status petitions were equally sampled. Assessments were administered using a tablet-based, audio-assisted computerized assessment in English and Spanish (for caregivers only). Additional study procedures are described in Authors, 2020 and Authors, 2021. All study procedures were approved by the Institutional Review Boards and Office for Human Research Protections at the University of California, San Francisco.

As described in detail previously (Authors, 2020), at baseline of the parent study, 401 youth-caregiver dyads were consented and followed for 24 months and completed assessments every 4 months (for a total of 7 time points). All youth (*n* = 313) and caregivers (*n* = 324) who participated in the 4-month follow-up assessment were offered the opportunity to separately consent/assent for the youth to complete additional (one-time) measures regarding past traumatic experiences; 273 youth completed these supplementary measures and of these youth, 271 also completed the 12-month follow-up assessment. Of these 271 youth, 262 responded to questions regarding expanded ACEs, so these youth and their caregivers comprise the sample for the current study. Youth demographics for this sub-study largely mirror those of the full parent study (Authors et al., 2020). Baseline and 4-month timepoints were selected based on when ACEs were first assessed in the parent study; the 12-month follow-up timepoint was selected to replicate and extend Authors (2021). The sample and procedures for the current report uses roughly the same sample and procedures described in depth in a prior longitudinal study of youth at first contact with the juvenile court (Authors, 2021, *N* = 271 with data on at least one domain of standard ACEs). The sample for the current report only includes the 262 dyads with data on expanded ACEs.

## Measures

**Demographics.** Standard demographic characteristics (e.g., gender, race, and ethnicity) for youth and caregivers were self-reported at baseline.

**Standard ACEs**. The 10 standard ACEs (Dong et al., 2004; Felitti et al., 1998) were assessed through a series of instruments administered at baseline and 4-month follow-up. Responses were operationalized such that exposure to each standard ACE included experiences at any point in the youth’s life up to and including the 4-month post-baseline assessment. Each individual ACE was coded as 1 = *yes* or 0 = *no*, accounting for endorsement of that ACE at baseline and/or 4-month follow-up (possible range = 0–10). For ACEs where both youth and caregiver report were available, an affirmative response by either reporter was considered endorsement of the ACE. Scores were prorated if youth had missing data for no more than two standard ACEs. Total number of ACEs was used, consistent with Authors, 2021 and other prior research among justice-impacted youth (e.g., Baglivio & Epps, 2016; Fox et al., 2015; Wolff et al., 2017). See Authors, 2021 for in depth descriptions of assessments and scoring.

***Abuse and neglect.*** Youth self-reported physical, emotional, and sexual abuse and physical and emotional neglect at 4-month follow-up with the Childhood Trauma Questionnaire Short-Form (CTQ-SF; Bernstein et al., 2003). The scale has good internal consistency, *w* = 0.80, 95% CI [0.65, 0.88]. Based on recommended *a priori* cutoff scores (low to moderate = 9–12; moderate to severe = 13–15; severe to extreme = > 15), youth with scores of nine or greater on any subscale were considered to have experienced that specific type of abuse or neglect (Marshall et al., 2013). One item from the Traumatic Life Events Inventory (TLE; Weathers et al., 2013) was also used to identify youth with exposure to sexual abuse (e.g., rape, attempted rape, made to perform any type of sexual act through force or threat of harm). Items are scored on a 5-point scale (0 = *happened to me*; 1 = *witnessed it*; 2 = *learned about it*; 3 = *not sure*; 4 = *doesn’t apply*); youth with a rating of 0 were considered to have experienced sexual abuse.

***Household dysfunction.****Caregiver substance use* was identified at baseline and 4-month follow-up using two caregiver-report items from the Parent Risk Behavior Assessment (Donenberg, Emerson, Bryant, Wilson, & Weber-Shifrin, 2001): (1) been told by a doctor they have a diagnosis of a substance use disorder; (2) been in treatment for drug and/or alcohol problems; and at 4-month follow-up using one youth-report item on the CTQ-SF (Bernstein et al., 2003): “My parents were too drunk or high to take care of the family.”

*Caregiver mental illness* was identified at baseline and 4-month follow-up using two caregiver-report items from the Parent Risk Behavior Assessment (PRBA; Donenberg, Emerson, Bryant, Wilson, & Weber-Shifrin, 2001): (1) been told by a doctor they have a psychiatric diagnosis; and (2) been in treatment for mental health difficulties.

*Exposure to domestic violence* was assessed at baseline and 4-month follow-up using a single item from the Adolescent Risk Behavior Assessment (ARBA; Donenberg et al., 2001) assessing whether youth had seen their “parent get pushed, slapped, hit, punched, or beat up by another parent, or their boyfriend or girlfriend?” (Donenberg et al., 2001).

*Caregiver separation or divorce* was identified at baseline and 4-month follow-up through caregiver report of current marital status on a demographic questionnaire, as well as youth report that participating caregiver was a stepparent.

*Caregiver incarceration* was assessed at baseline and 4-month follow-up via a single item on the Parent Arrest and Treatment History questionnaire (Authors, 2020) assessing whether caregivers had ever been incarcerated.

**Expanded ACEs.** The five expanded ACEs were assessed through a series of instruments administered at baseline and 4-month follow-up. For those assessed at both timepoints, responses were operationalized such that exposure to each expanded ACE included experiences at any point in the youth’s life up to and including the 4-month post-baseline assessment. Each individual ACE was coded as 1 = *yes* or 0 = *no*, accounting for endorsement of that ACE at baseline and/or 4-month follow-up (possible range = 0–5). Scores were prorated if youth had missing data for no more than 1 expanded ACE.

*Witnessing violence* was assessed at the 4-month follow-up using five items from the TLE (Weathers et al., 2013). Youth who responded they had witnessed physical assault, assault with a weapon, sexual assault, or sudden violent death, were considered to have exposure to this ACE.

*Discrimination based on race or ethnicity* was assessed at baseline using the 11-item Everyday Discrimination Scale (EDS; Williams et al., 1997). The scale has excellent internal reliability, *w* = 0.93, 95% CI [0.91, 0.94]. Youth self-reported their experiences with discrimination in their day-to-day life on 10 items (scale from 1 = *almost every day* to 6 = *never*). Items were reverse scored and summed to create a total score, with higher values reflecting more frequent experiences with discrimination. Youth also responded to one item identifying what they thought was the main reason for these experiences with discrimination. Youth who endorsed experiencing discrimination a few times a year or more (on any item) and who identified the source of discrimination being due to their ancestry or national origin, race, or their shade of skin color, were considered to have exposure to discrimination.

*Adverse neighborhood experience* was assessed at baseline using the 6-item neighborhood disadvantage subscale from the Neighborhood Environment Scale (NES; Crum et al., 1996; Lang et al., 2010). Items are rated as 1 = *true* or 0 = false and then summed to yield an overall score; higher scores reflect higher levels of neighborhood disadvantage. The neighborhood disadvantage subscale at baseline had good reliability *w* = 0.87, 95% CI [0.84, 0.89].

*Experience with bullying* was assessed at baseline using a single item asking youth if they had ever been bullied (yes/no).

*Placement in foster care* was assessed at baseline and the four-month follow-up. Youth and caregivers both responded to two items assessing whether the youth had ever: (1) been removed from the home by the Department of Children, Youth, and Families or another state child welfare agency; and (2) been in therapeutic foster care (defined to families as *“where foster parents have been trained to provide care”)*.

**Youth behavioral health outcomes.** Behavioral outcomes for youth were assessed at 4-and 12-month follow-up using standardized measurement with validated tools.

***Alcohol and cannabis use.*** Youth self-reported substance use was assessed at months 4 and 12 using the Adolescent Risk Behavior Assessment (ARBA; Donenberg et al., 2001). Alcohol and cannabis use were each rated dichotomously (0 = *no use*, 1 = *any use*) over the prior 120 days.

***Alcohol- and cannabis-related consequences.*** Youth self-reported substance use consequences were assessed at 12-month follow-up with two measures: (1) Brief Young Adult Alcohol Consequences Questionnaire (Kahler et al., 2005), a 24-item measure; and (2) Brief Marijuana Consequences Scale (Simons et al., 2012), a 21-item measure. Items endorsed on each scale were rated 1 = *yes* or 0 = *no* and summed to create a total score; higher scores indicate greater use-related consequences.

***Youth internalizing and externalizing symptoms***. Youth internalizing symptoms and externalizing symptoms were assessed at the 4- and 12-month follow-ups using the Behavior Assessment System for Children, Second Edition (BASC-2; Reynolds & Kamphaus 2006). Youth reported internalizing symptoms on the Internalizing composite scale, a broad index of inwardly directed distress; items are rated on 2-point true or false responses and 4-point scales where 1 = *never* and 4 = *almost alway*s. Caregivers reported on youth externalizing symptoms on the Externalizing composite scale, a broad index of disruptive behavior problems such as aggression, hyperactivity and delinquency; items are rated on 4-point scales (1 = *never* and 4 = *almost alway*s). Summed raw scores were converted to a T-score (standardized scores with a mean of 50 and standard deviation of 10) based on a general adolescent sample.

***Youth trauma symptoms.*** Post-traumatic stress symptoms were assessed via youth self-report at months 4 and 12 with the 9-item National Stressful Events Survey PTSD Short Scale (NSESSS; Kilpatrick et al., 2013). The scale has excellent internal consistency at both month 4, *w* = 0.95, 95% CI [0.93, 0.96], and month 12, *w* = 0.98, 95% CI [0.97, 0.98]. Higher scores reflect greater severity of PTSD. Items were rated on a scale from 0 (not at all) to 4 (extremely); each item contained a response option, “I have never experienced a stressful event.” If a youth responded to more than two items with “I have never experienced a stressful event,” entire scales were recoded as missing. When up to two items were left unanswered, prorated scores ((sum of items answered x total number of items on measure)/number of items answered, rounded to nearest whole number) were calculated. Prorated scores were also calculated when youth answered, “I have never experienced a stressful event” (coded as missing) to up to two items, only if they endorsed at least one abuse ACE (*n* = 17).

## Analytic Approach

Analyses were conducted using SPSS and R (R Core Team, 2021) statistical software. Preliminary analyses included descriptive statistics and bivariate correlations between all study variables. Missing data ranged from 0–33.6%, with greater missingness on 12-month outcomes (14.1–20.2% except for trauma symptoms, which was 33.6%). Little’s MCAR test was significant (*X*^2^(261) = 316.64, *p* = .010), indicating data were not missing completely at random. As such, we employed multiple imputation for the missing data using the *mice* version 3.14.0 R package (van Buuren & Groothuis-Oudshoorn, 2011); model coefficients were pooled from 100 imputed datasets via a maximum of 20 iterations. Regression models from each imputed dataset were pooled via the mice (Van Burren & Groothuis-Oudshoorn, 2011) package’s *pool*() function. The mice-compatible the *how_many_imputations*() function confirmed that 100 imputations was sufficient for all regression analyses (range of necessary imputations = 13 to 50) to achieve a desired standard error of 0.05 (von Hippel, 2020).

Mann-Whitney U Tests were used to examine differences in number of expanded ACEs based on gender and whether youth had an open status or delinquent petition. A Kruskall-Wallis test was used to examine differences in number of expanded ACEs based on race and ethnicity. Differences in number of standard ACEs based on these demographic factors have been previously reported (Authors, 2021).

Two independent variables were used to examine the impact of ACEs on behavioral health outcomes: (1) the standard ACEs score, which is the sum of the ten standard ACEs (including responses from baseline and 4-month follow-up assessments; possible range = 0–10); and (2) the expanded ACEs score, which is the sum of the expanded ACEs (including responses from baseline and 4-month follow-up assessments; possible range = 0–5). Via the MASS package version 7.3–54 (Venables & Ripley, 2002), regression analyses were used to examine the associations between standard and expanded ACEs with 12-month follow-up youth behavioral health outcomes. Linear regressions were used for continuous outcomes (psychiatric symptoms), negative binomial for count outcomes (consequences of substance use), and logistic regressions for dichotomous outcomes (alcohol and cannabis use). All regression analyses co-varied youth age, gender, and ethnicity (i.e., Latinx vs. non-Latinx), as well as prior substance use or psychiatric symptoms (e.g., controlling for past 4-month alcohol use at 4-month follow-up when examining past 4-month alcohol use at 12-month follow-up as outcome). Analyses with internalizing and externalizing symptoms as the outcome did not co-vary age, because t-scores are determined with age-based norms. Due to small subgroup sizes within the Black, multiracial, and other non-Latinx groups, and the lack of significant differences in outcomes based on race and ethnicity (Authors, 2021), primary analyses used a binary indicator of whether youth identified as Latinx or not. For each regression model, Model 1 included the standard ACEs score as an independent variable and Model 2 added the expanded ACEs score. Multivariate Wald tests were used to examine whether models with the expanded ACEs improved model fit compared to models with only the standard ACEs score.

## Results

### Descriptive Statistics and Bivariate Relationships

Youth (*N* = 262) were on average 14.5 years old (*SD* = 1.6), 51.9% boys, and self-identified as 42.1% Latinx, 34.9% white non-Latinx, 8.4% Black non-Latinx, 8.4% other non-Latinx, and 6.1% Multiracial non-Latinx. Of the 110 youth who identified as Latinx, youth identified their main ethnic origin as 55.5% Puerto Rican, 28.2% Dominican, 3.6% Guatemalan, 1.8% Columbian, 0.9% Bolivian, 0.9% Cuban, 0.9% Mexican, 0.9% Panamanian, 1.8% mixed Latinx, 0.9% unspecified other Latinx, and 1.8% did not specify. Half (48.9%) had an open delinquency petition and half (51.1%) an open status petition with the court. At baseline, 36.3% of JIY reported lifetime use of alcohol and 51.4% reported lifetime use of cannabis. Regarding recent use, 30.3% and 24.9% reported alcohol use in the past 4 months at the 4- and 12- month follow-ups, respectively; 41.4% and 37.5% reported past 4-month cannabis use at the 4- and 12- month follow-ups, respectively. Youth reported low levels of consequences associated with alcohol (*M* = 0.68, *SD* = 2.8) and cannabis (*M* = 0.99, *SD* = 2.8) use at 12-month follow-up. On average, levels of youth internalizing (4-month: *M* = 52.6, *SD* = 15.0; 12-month: *M =* 50.6, *SD* = 14.8) and externalizing (4-month: *M* = 58. 0, *SD* = 13.4; 12-month: *M =* 56.1, *SD* = 13.0) symptoms were within normal range, and average trauma symptoms (4-month: *M* = 7.8, *SD* = 9.0; 12-month: *M =* 6.4, *SD* = 9.2) were relatively low.

**Fig. 1:**
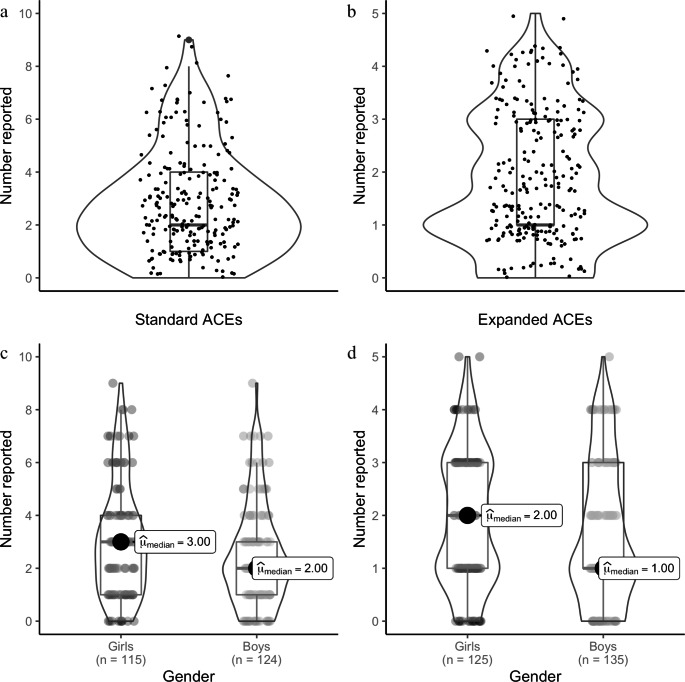
Box plots depict distribution of standard (a) and expanded (b) adverse childhood experiences (ACEs) reported by the full sample (N = 262). Violin plots depict distribution of standard ACEs reported by girls (n = 115) and boys (n = 124) (c), and distribution of expanded ACEs reported by girls (n = 125) and boys (n = 135) (d)

The majority (89.2%) of youth experienced at least once ACE, endorsing an average of three standard ACEs (SD = 2.0; range 0 to 9) by the 4-month follow-up; this includes on average, one ACE (SD = 1) in each standard category (abuse, neglect, household dysfunction) (Authors, 2021). Most youth (83.2%) also experienced at least one expanded ACE, endorsing an average of two expanded ACEs (SD = 1.3, range 0 to 5) by the 4-month follow-up; 29.2% had witnessed violence, 17.3% had been in foster care, 42.7% had been bullied, 14.8% experienced racial discrimination, and 60.8% lived in a disadvantaged neighborhood. Girls endorsed significantly more expanded ACEs (median = 2) than boys (median = 1), *W*(260) = 9668.00, *p* = .04; similar gender differences were found in regards to girls reporting significantly more standard ACEs in our prior report (Authors, 2021). There were no significant differences in number of standard (Authors, 2021) or expanded ACEs (*W*(262) = 9655.50, *p* = .07) based on whether youth had a first-time status or delinquent petition. Regarding race and ethnicity, multi-racial non-Latinx youth endorsed significantly more expanded ACEs than non-Latinx white and Latinx youth (*H* = 9.95, *p* = .04); however, these differences were not significant when adjusted by a Bonferroni correction. Figure 1 displays the distribution of the ACEs scores.

## Relationship Between ACEs and Youth Behavioral Health Outcomes

At the bivariate level, expanded ACEs were significantly associated with 4-month follow-up internalizing (*r* = .36, *p* < .001), externalizing (*r* = .14, *p =* .04), and trauma (*r* = .34, *p* < .001) symptoms, as well as 12-month follow-up internalizing (*r* = .30, *p* < .001) symptoms. After adjusting for sociodemographic factors, prior use, and the expanded ACEs score, standard ACES predicted cannabis-related consequences (*B* = 0.09, *SE* = 0.02 95% CI *=* 0.00 − 0.09 *p* = .04*).* Standard and expanded ACEs were not related to any other behavioral health outcomes. Results of multivariate Wald tests indicated that models with standard and expanded ACEs did not improve model fit compared to models with only the standard ACEs score. Findings are displayed in Tables 1 and 2.

**Table 1:** *Association between adverse childhood experiences and youth substance use at 12 month follow-up* (*N* = 262)

	Alcohol Use				Cannabis Use				Alcohol Consequences				Cannabis Consequences			
Variable	B	SE	OR	95% CI	B	SE	OR	95% CI	B	SE	95% CI	β	B	SE	95% CI	β
**Model 1: Standard ACEs**																
Age	0.13	0.12	1.14	0.89, 1.46	0.25*	0.12	1.28	1.02, 1.61	0.03	0.03	-0.02, 0.08	0.06	0.07*	0.03	0.01, 0.13	0.11
Latinx	-0.21	0.36	0.81	0.39, 1.66	-0.67^+^	0.34	0.51	0.26, 1.01	-0.05	0.07	-0.19, 0.10	-0.05	-0.05	0.08	-0.22, 0.11	-0.05
Prior Use	1.88***	0.39	6.56	3.05, 14.12.	2.00***	0.35	7.39	3.67, 14.88	0.30***	0.09	0.12, 0.47	0.32	0.33***	0.09	0.14, 0.51	0.33
Gender	0.18	0.36	1.20	0.59, 2.46	-0.59^+^	0.34	0.56	0.28, 1.09	0.00	0.07	-0.15, 0.14	-0.02	-0.07	0.09	-0.24, 0.09	-0.07
ACEs (standard)	0.15 ^+^	0.09	1.16	0.98, 1.38	0.07	0.08	1.07	0.91, 1.26	0.03^+^	0.02	0.00, 0.07	0.00	0.04^+^	0.02	-0.001, 0.09	0.08
Constant	-4.31*	1.81	0.01	0.00, 0.48	-4.78**	1.66	0.01	0.00, 0.22	-0.46	0.36	-1.16, 0.25	0.14	-0.85	0.41	-1.65, -0.05	0.24
**Model 2: Standard and Expanded ACEs**																
Age	0.16	0.13	1.18	0.92, 1.51	0.23*	0.12	1.26	1.00, 1.59	0.03	0.25	-0.02, 0.08	-0.05	0.06*	0.03	0.01, 0.12	0.10
Latinx	-0.18	0.37	0.84	0.40, 1.73	-0.69*	0.35	0.50	0.25, 1.00	-0.05	0.07	-0.19, 0.10	-0.05	-0.05	0.08	-0.2, 0.11	-0.05
Prior Use	1.95***	0.40	7.05	3.21, 15.47	2.05***	0.36	7.73	3.79, 15.77	0.30**	0.09	0.12, 0.47	0.30	0.34***	0.09	0.15, 0.52	0.34
Gender	0.21	0.37	1.23	0.60, 2.53	-0.60^+^	0.34	0.55	0.28, 1.08	-0.01	0.07	-0.15, 0.14	-0.01	-0.08	0.09	-0.25, 0.09	-0.08
ACEs (standard)	0.12	0.09	1.12	0.94, 1.34	0.08	0.09	1.09	0.92, 1.29	0.04	0.02	-0.004, 0.07	0.07	0.05*	0.02	0.002, 0.09	0.09
ACEs (expanded)	0.28 ^±^	0.15	1.32	0.99, 1.76	-0.12	0.14	0.89	0.68, 1.17	-0.01	0.03	-0.07, 0.05	-0.01	-0.04	0.03	-0.10, 0.04	-0.04
Constant	-5.20**	1.89	0.01	0.00, 0.23	-4.38*	1.70	0.01	0.00, 0.36	-0.43	0.36	-1.14, 0.28	0.14	-0.74	0.42	-1.56, 0.08	0.24

*Note.*^+^*p* < .10; ^*^p < .05; ^**^p < .01; ^***^p < .001; ACEs = adverse childhood experiences; Model 1 vs. Model 2 comparisons: Alcohol Use χ^2^ (1, 228.24) = 3.61, *p* = .059; Cannabis Use χ^2^ (1, 230.38) = 0.74, *p* = .390; Alcohol Consequences χ^2^ (1, 235.45) = 0.11, *p* = .75; Cannabis Consequences χ^2^ (1, 235.04) = 0.97, *p* = .33

**Table 2:** *Association between adverse childhood experiences and youth psychiatric symptoms* (*N* = 262)

	Internalizing symptoms				Externalizing symptoms				Trauma symptoms			
Variable	B	SE	95% CI	β	B	SE	95% CI	β	B	SE	95% CI	β
**Model 1: Standard ACEs**												
Age	-	-	-	-	-	-	-	-	-0.74*	0.36	-1.46, -0.02	-0.13
Latinx	-0.75	1.68	-4.07, 2.57,	-0.75	-0.23	0.98	-2.17, 1.71	-0.23	-1.00	1.18	-3.32, 1.32	-0.11
Prior Symptoms	0.52***	0.06	0.40, 0.64	0.52	0.80***	0.04	0.73, 0.87	0.80	0.47***	0.08	0.32, 0.62	0.46
Gender	-1.67	1.71	-5.04, 1.69	-1.67	2.25*	1.02	0.25, 4.26	2.25	-0.34	1.26	-2.82, 2.14	-0.04
ACEs (standard)	0.52	0.45	-0.36, 1.41	1.06	0.21	0.26	-0.29, 0.72	0.42	0.52	0.32	-0.11, 1.16	0.11
Constant	22.43***	3.49	15.54, 29.32	23.86	7.87***	2.17	3.59, 12.14	8.44	12.64	5.29	2.20, 23.08	0.06
**Model 2: Standard and Expanded ACEs**												
Age	-	-	-	-	-	-	-	-	-0.79*	0.37	-1.51, -0.06	-0.14
Latinx	-0.67	1.68	-3.98, 2.64	-0.67	-0.23	0.99	-2.18, 1.71	-0.24	-1.04	1.18	-3.37, 1.29	-0.11
Prior Symptoms	0.50***	0.06	0.37, 0.62	0.50	0.80***	0.04	0.73, 0.87	0.80	0.49***	0.08	0.34, 0.65	0.48
Gender	-1.64	1.70	-4.99, 1.71	-1.64	2.24*	1.01	0.23, 4.24	2.24	-0.36	1.26	-2.85, 2.13	-0.04
ACEs (standard)	0.45	0.45	-0.43, 1.34	0.92	0.22	0.27	-0.31, 0.74	0.44	0.58	0.33	-0.08, 1.23	0.13
ACEs (expanded)	1.05	0.69	-0.31, 2.40	1.36	-0.06	0.41	-0.88, 0.76	-0.08	-0.54	0.49	-1.51, 0.42	-0.08
Constant	22.23***	3.47	15.38, 29.08	25.32	7.93**	2.21	3.58, 12.28	8.41	14.03**	5.39	3.38, 24.67	0.07
**Model Comparison**	χ^2^	df1, df2	*p*		χ^2^	df1, df2	*p*		χ^2^	df1, df2	*p*	
Model 1 vs. Model 2	2.32	1, 224.54	0.13		0.02	1, 198.24	0.88		1.22	1, 200.30	0.27	

*Note.*^+^*p* < .10; ^*^p < .05; ^**^p < .01; ^***^p < .001; ACEs = adverse childhood experiences; models for internalizing and externalizing do not include age as a covariate because the *t*-score outcome already accounts for age; Internalizing model 1 Adjusted R2 = 0.34 [95% CI = 0.24, 0.44]; Internalizing model 2 Adjusted R2 = 0.35 [95% CI = 0.24, 0.45]; Externalizing model 1 Adjusted R2 = 0.71 [95% CI = 0.64, 0.77]; Externalizing model 2 Adjusted R2 = 0.71 [95% CI = 0.64, 0.77]; Trauma symptoms model 1 = Adjusted R2 = 0.27 [95% CI = 0.15, 0.39]; Trauma symptoms model 2 = Adjusted R2 = 0.27 [95% CI = 0.15, 0.40]

## Discussion

A growing body of research has documented a link between the standard ACEs and behavioral health needs among JIY (Folk et al., 2021); however, less attention has been paid to the expanded ACEs, which capture social and community level adversity. The current study examined the prevalence of the expanded ACEs and their prospective association, above and beyond the standard ACEs, and behavioral health outcomes among youth at time of first contact with the juvenile court.

Study findings reveal that the majority of youth coming into first-time contact with the juvenile court have already experienced multiple ACEs, including on average three standard and two expanded ACEs. High rates of exposure to the expanded ACEs were found, and in particular, high rates of neighborhood disadvantage, bullying, and witnessing violence. Girls endorsed more expanded ACEs than boys, and no differences were found based on race, ethnicity or whether youth had an open status vs. delinquent petition.

In multivariate analyses accounting for standard ACEs, the expanded ACEs were unrelated to the behavioral health outcomes assessed. After accounting for the expanded ACEs in multivariate models, the standard ACEs score predicted cannabis-related consequences. These findings are in part consistent with studies documenting a link between ACEs and substance use among JIY (Benedini & Fagan, 2020; Chatterjee et al., 2018; Scheidell et al., 2018). The current findings also suggest that even accounting for the expanded ACEs in addition to the standard ACEs, predictive utility for the behavioral health outcomes examined is very limited.

The current findings also diverge from our prior analysis in an important way. In our earlier analysis (Authors, 2021) youth who reported a greater number of standard ACEs were more likely to use alcohol and to experience higher levels of posttraumatic stress symptoms during the year following first court contact. This original analysis used listwise deletion; however, in the current models with imputed data (which improve power to detect statistical effects), standard ACEs were not significantly related to posttraumatic stress symptoms or alcohol use. These alternative findings may point to the need to consistently utilize rigorous imputation strategies in future ACEs studies. For example, using 100 imputed datasets with more stable standard error estimates and more statistical power due to less missing data led to different results than our previous analyses, including those using a smaller number (i.e., 10) of imputed datasets.

## Strengths, Limitations, and Future Directions

Strengths of the current study include the prospective design, use of empirically validated assessments, multi-informant approach, and examination of a wider range of ACEs than most prior studies with JIY. These are among the first data to examine the association between expanded ACEs and behavioral health in JIY.

The parent study’s primary aims were not centered on trauma, and as such, there are several limitations to the assessment of ACEs in the current study. We triangulated across multiple assessments to determine exposure to ACEs rather than using a single ACEs questionnaire, and limitations include: (1) the household dysfunction ACEs of caregiver substance use, mental illness, and incarceration only capture information about the participating caregiver and not all primary caregivers in the youth’s life; (2) to reduce participant burden, bullying was assessed using a single binary item rather than asking about experiences with specific bullying behavior, which may yield different estimates; (3) witnessing violence, as assessed by the TLE, does not distinguish between violence in the home and in the community, and it is therefore possible that some youth who witnessed violence only in the home were double-counted in the witnessing violence and domestic violence ACEs; (4) caregiver separation was determined based on the participating caregiver’s marital status and relationship to the youth, and it is possible no prior divorce or separation had occurred when the caregiver identifies as a step-parent. Regarding timing of ACEs assessment, abuse and neglect were only assessed at the 4-month follow-up as part of a sub-study. Relatedly, some expanded ACEs were only assessed at baseline and subsequently at 8-month intervals; only baseline reports were used for these expanded ACEs. It is possible we failed to account for youth who experienced these expanded ACEs for the first time between baseline and the 4-month follow-up (when other standard ACEs were captured), however the specific timing of ACEs exposure is unknown. Future studies with JIY should assess exposure, including age of exposure, to both standard and expanded ACEs, at first-time juvenile legal system contact in order to understand both how legal system contact may impact ACEs and differences in predictive utility depending upon age of ACEs exposure. Future studies should also consider assessing ACEs using a single measure.

Second, JIY in this study were recruited from one family court in the northeastern U.S. and may not be representative of all youth at first juvenile court contact. Future research is needed to determine whether findings generalize to other U.S. jurisdictions and internationally. Third, our models did not account for whether youth received behavioral health treatment during the follow-up period, which may moderate the association between ACEs and the behavioral health outcomes examined; it is possible youth were referred to treatment at this point of first court contact, which may have been successful in mitigating adverse outcomes, particularly for those with high ACE scores. This is an important direction for future research. Finally, we may have been underpowered to detect small yet clinically meaningful associations given the relatively limited sample size. Sample size also limited our ability to conduct more nuanced analyses of differences in the association between ACEs and behavioral health outcomes among youth from different racial and ethnic groups. Further, our self-report measure of ethnicity was limited to Latinx origin and did not capture the full range of ethnic origins for participants; this limited our analyses to the binary variable of Latinx vs. non-Latinx.

## Practical Implications

Findings suggest limitations to using expanded ACEs as a clinical indicator of future behavioral health treatment need, as the expanded ACEs did not predict the behavioral health outcomes examined in this sample of first-time JIY. Although studies suggest screening for ACEs is useful in predicting adverse mental health and physical health outcomes on the population level, predictiveness of ACE scores for health outcomes on the individual level is low (Baldwin et al., 2021) and the current state of research does not support use of ACEs screening (a population-based research tool) for individual level clinical decision making (Anda et al., 2020). Of note, the ACEs score does not account for intensity, chronicity, frequency, or developmental timing of trauma exposure. Two youth can have the same ACEs score with very different experiences; for example, a youth who experienced an amicable parental separation during their adolescence would receive the same score as a youth who was sexually abused repeatedly throughout childhood. Further, protective factors that influence biological stress responses are not captured in the ACEs score. These limitations may explain the lack of predictive utility of the expanded ACEs score among our sample of JIY, even when including the relevant expanded ACEs. These limitations also highlight future research directions, which include generating and testing theory on how ACEs contribute to and maintain downstream behavioral health symptoms in JIY populations. Future studies may also seek to understand differential pathways through which eco-developmental theoretical levels contribute to behavioral health symptoms.

Further, although our findings highlight the limited utility of the expanded ACEs as a screening tool for behavioral health diagnoses or need for services, it is possible that assessment of expanded ACEs may be useful for understanding histories of adversity in JIY to contextualize assessment and care. Assessment of ACEs may also be component of trauma-informed care, which targets systems that generate and maintain trauma vs. psychiatric symptoms and has been shown to be efficacious with JIY across a range of behavioral health outcomes (Zettler, 2021). Future work is needed to understand how ACEs may be systematically incorporated into trauma-informed care approaches for JIY populations.

Finally, these findings suggest JIY with a history of ACEs may benefit from screening and referral to substance use treatment, particularly for cannabis use. First court contact is a critical window for this screening and referral to occur, as addressing behavioral health needs prior to escalation to substance use disorder and further system entrenchment is key.

## Conclusion

Youth who come into contact with the juvenile legal system experience high rates of adversity, including multiple standard and expanded ACEs. These youth also commonly present with complex behavioral health needs upon first contact with the court, and screening and referral to treatment at this entry point into the legal system is crucial. Results from the current study do not support the use of ACEs as an indicator of behavioral health treatment need; further research into the effects of exposure to the social and community level adversities captured by the expanded ACEs is needed before this can be used as an indicator of behavioral health treatment need in clinical practice.
